# Clinical results of the open ring PMMA guider assisted capsulorrhexis in cataract surgery

**DOI:** 10.1186/s12886-018-0782-6

**Published:** 2018-05-10

**Authors:** Jee Hye Lee, Yong Eun Lee, Choun-Ki Joo

**Affiliations:** 1 Department of Ophthalmology & Visual Science, Seoul St. Mary’s Hospital, The Catholic University of Korea, Seoul, South Korea; 2 The Ian eye center, Seoul, South Korea

**Keywords:** Continuous curvilinear capsulorrhexis(CCC), Open ring-shaped guider for CCC(ORGC), Ideal CCC

## Abstract

**Background:**

To compare the results of continuous curvilinear capsulorrhexis(CCC) after application of an open ring-shaped guider compared with a free-hand procedure in eyes with cataracts.

**Methods:**

This study comprised patients undergoing cataract surgery in Seoul St.Mary’s Hospital, The Catholic University of Korea. Eyes were grouped depending on the capsulotomy method; CCC was performed by free-hand procedure on 94 eyes (free-hand group), and it was performed under the guidance after introduction of an open ring-shaped guider on consecutive 89 eyes (guided group). Horizontal and vertical diameter, area and circularity of capsulotomy were measured postoperatively at one day, two months and six months. Differences in parameters and the percentage of ideal capsulorrhexis were analyzed between the two groups.

**Results:**

On the first postoperative day, the vertical diameter in the guided group (5.24 ± 0.16 mm) was significantly longer than that of the free-hand group (5.01 ± 0.65 mm, *P* = 0.019). The area of capsulotomy was larger in the guided group (21.55 ± 0.87 mm^2^) than that of the free-hand group (20.34 ± 2.96 mm^2^, *P* < 0.001). Circularity in the guided group (0.84 ± 0.03), was significantly greater than that of the free-hand group (0.69 ± 0.17, *P* = 0.036). Ideal capsulorrhexis was obtained in 60 eyes (67%) in the free-hand group and 81 eyes (86%) in the guided group.

**Conclusions:**

After introduction of an open ring-shaped guider, CCC became larger and more circular with less anterior capsular contracture. The rate of acquiring ideal capsulorrhexis was higher in the guided group than it was in the free-hand group for six months after surgery.

## Background

Continuous curvilinear capsulorrhexis (CCC) is a standard technique in cataract surgery that is preferable to the can-opener capsulotomy [1]. CCC is essential for the safety of phacoemulsification and intraocular lens (IOL) implantation because it permits safe hydrodissection, cortical cleanup, and IOL centration while preventing posterior capsule opacification (PCO) [2, 3].

Previous studies suggest that the anterior capsulotomy size and circularity are important. If the capsulotomy is too small, fibrosis and hyperopic shift may ensue [4]. If the capsulotomy is too large or asymmetric, the IOL may be adversely affected by tilt, rotation, decentration, or posterior capsular opacification [5].

The ideal capsulorrhexis is a well-centered opening that perfectly overlaps the IOL optic by 360° [6]. This alignment ensures that the IOL contained in the capsular bag is close to the effective lens position (ELP) to avoid an inaccurate IOL power calculation [7]. When the capsular bag is close to the ELP, it prevents optic tilt, decentration, myopic shift, and capsular opacification due to symmetric contractile forces on the capsular bag that cause a shrink-wrap effect. Newer IOLs, including toric-, multifocal- and accommodating IOLs, are more sensitive to accurate positioning and would benefit from more reproducible sizing, shaping, and centration of the anterior capsulotomy.

Several methods that facilitate CCC completion have been invented. One of the widely used instruments is Wallace’s circular corneal marker [8]. But this device provided only a rough guide outside the cornea. To resolve this problem, Suzuki et al. designed a marker that makes a semicircular mark directly on the lens capsule [9]. However, this marker was difficult to manipulate because of its metallic material and fixed semicircular design. The caliper proposed by Tassignon et al. is difficult to insert through a small corneal incision under 3.0 mm [10]. The VERUS ophthalmic caliper (Mile High Ophthalmics, Denver, CO) is a ring-shaped silicone device and has the enhanced lateral stability with micropatterning [11]. Also, Zepto precision pulse capsulotomy (Mynosys, Fremont, CA) which creates a capsulotomy automatically has been introduced and available on the market [12].

**Fig. 1:**
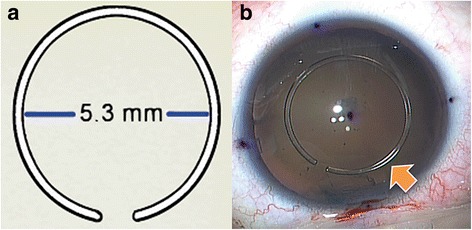
**a** Open ring guider for CCC; inflexible polymethyl methacrylate caliper ring with an internal diameter of 5.3 mm. It is easy to insert into the eye because of its open-ring shape. **b** Open ring-shaped guider for continuous curvilinear capsulorrhexis (ORGC) (arrow) is inserted into the anterior chamber

Recently, we reported a surgical technique using a new transparent open-ring guider made of PMMA to make a round, precise CCC with less radial tear [13]. Briefly, the open ring-shaped guider for CCC (ORGC, Lucid Co., Seoul, Korea) is a ring-shaped ruler with arc of 10° when opened. The ORGC is 0.125 mm thick with an internal diameter of 5.3 mm and an outer diameter of 5.8 mm (Fig. 1). It acts as a visual guide during the capsulorrhexis.

The purpose of our study was to compare the outcomes of CCC after application of an open ring-shaped guider compared with a free-hand procedure in eyes with cataracts.

## Methods

This study was conducted by retrospective chart review from patients who had uneventful phacoemulsification and intraocular lens implantation. All of the surgeries were performed by the same surgeon (C-K.J) between 2012 and 2013 at Seoul St. Mary’s Hospital. Written informed consent was obtained from all of the patients before their records were used. The study complied with the institutional review board regulations at Seoul St. Mary’s Hospital (CMC clinical research coordination center, study approval number: KCI2DISE0320), informed consent regulations, and the Declaration of Helsinki.

CCC was performed by free-hand procedure on 94 eyes (free-hand group). After introduction of an open ring-shaped guider, capsulorrhexis was performed under the guidance on consecutive 89 eyes (guided group). Each patient underwent complete ophthalmologic evaluation before surgery. Exclusion criteria included previous ocular surgery, trauma, active ocular disease which would affect post-operative visual acuity, poor pupil dilation, poor red reflex, or known zonule weakness.

The cataract severity was evaluated based on nuclear opacity using the lens opacities classification system (LOCS III). The axial length and K readings were measured using optical biometry (IOL Master, Carl Zeiss Meditec AG, Germany) before surgery. Phacoemulsification was performed under topical anesthesia with 4% lidocaine and 0.5% proparacaine hydrochloride (Alcaine; Alcon Laboratories, USA). A 2.2 mm clear corneal incision was made, and 1.4% sodium hyaluronate (Healon GV®; Advanced Medical Optics, USA) was injected into the anterior chamber. In the guided group, the surgeon picks up one end of the guider with forceps and turns the ORGC clockwise to insert it into the anterior chamber gently. After the guider is placed on the anterior capsule, the surgeon performed CCC along the internal border of the guider. In the free-hand group, without guidance, the surgeon made a round CCC targeted at a 5.3 mm diameter. Then the IOL forceps was used to remove the guider in a counterclockwise fashion through the corneal incision. Inserted IOLs were single-piece, monofocal, aspheric, hydrophobic acrylic lenses with 6 mm optic diameter (total diameter 13 mm) and 5° angulation (EC-1 YH PAL®, Aaren Scientific, Inc., USA).

Digital photographs under retro-illumination were used to analyze the size of capsulotomies. Photographs were taken during surgery, immediately after the CCC procedure, and postoperatively at 1 day, 2 months, and 6 months. Image J (National Institute of Health, Bethesda, MD, USA) was used to measure the diameter, area, and circularity of the images. The diameters of the capsulotomies were measured in relation to the incision site. The horizontal diameter refers to the diameter along the same axis as the incision (if a temporal incision was made, this would be nasal to temporal direction) and the vertical diameter is perpendicular to that axis. Circularity was calculated to determine if the capsulotomy shape was regular according to the following formula:

$$ {f}_{\mathrm{circ}}=\frac{4\uppi A}{P^2} $$

(A = area, P = perimeter)

A circularity value of 1.0 indicates a perfect circle [14]. The percentage of ideal capsulorrhexis was also determined. Ideal capsulorrhexis is defined by a capsulotomy opening that completely overlaps the edge of the IOL optic at postoperative day one.

All data were analyzed using SPSS software (IBM SPSS Statistics version 19.0, Inc. Chicago, IL, USA). Student t-test and Chi-square test were used to compare the baseline characteristics of two groups. Mann-Whitney tests and Chi-square tests were used to compare the horizontal diameter, vertical diameter, area and circularity of the capsulotomy in the two groups. The significance level was set at *P* < 0.05 in all statistical analyses.

## Results

A total of 183 eyes were assessed, with 89 eyes in the guided group and 94 eyes in the free-hand group. Pre-operative mean axial length and k reading were 23.98 ± 2.76 mm, 46.56 ± 1.89 D in the free-hand group and 24.16 ± 2.48 mm, 44.16 ± 1.44 D in the guided group. Nuclear opacity grade was 2.98 ± 0.86 in the free-hand group and 3.14 ± 0.93 in the guided group. The differences between the two groups were not statistically significant with regard to age, sex, axial length, mean K reading, and cataract severity before surgery (Table 1). Patients tolerated the surgery well and there were no intra- or postoperative complications. After 6 months, mean BCVA was LogMAR 0.36 ± 0.25 in free-hand group and LogMAR 0.31 ± 0.29 in guided group (*P* = 0.14).

**Table 1:** Demographics of patients

Demographic	Free-hand group (*n* = 94)	Guided group (*n* = 89)
Age (y)	63.80 ± 9.48	64.08 ± 10.75
Sex (M:F)	43:51	41:48
Axial length (mm)	23.98 ± 2.76	24.16 ± 2.48
Inserted IOL	One-piece hydrophobic acrylic lens(EC-1YH PAL®)	
Mean K reading	46.56 ± 1.89	44.16 ± 1.44
Nuclear opacity (NO, LOCS III)	2.98 ± 0.86	3.14 ± 0.93

*NO* nuclear opalescence, *LOCS* lens opacities classification system

Table 2 shows the mean and standard deviation values of capsulorrhexis parameters measured at 1 day postoperatively. The capsulotomies were not perfectly round and were slightly different from the intended diameter of 5.3 mm. The vertical diameter in the guided group was significantly longer than that in the free-hand group (*P* = 0.019), but the horizontal diameter was not significantly different across the two groups (*P* > 0.05). As a result, the area of capsulotomy (mm^2^) was significantly larger in the guided group (*P* < 0.001). The circularity in the guided group was significantly better than in the free-hand group (*P* = 0.036). The percentage of ideal capsulorrhexis was 67% in the free-hand group and 86% in the guided group, which is statistically significant (*P* = 0.011).

**Table 2:** Comparison of size, circularity, and rate of ideal capsulorrhexis postoperatively at 1 day

Parameter	Free-hand group	Guided group	*P* value
Parallel diameter (mm)	5.17 ± 0.40	5.24 ± 0.21	> 0.05*
Perpendicular diameter (mm)	5.01 ± 0.65	5.24 ± 0.16	0.019*
Area of capsulotomy (mm ^2^)	20.34 ± 2.96	21.55 ± 0.87	< 0.001*
Circularity of capsulotomy	0.69 ± 0.17	0.84 ± 0.03	0.036*
Ideal capsulorrhexis (%)	67	86	0.011†

*Mann-Whitney U test

†Chi-square test

**Fig. 2:**
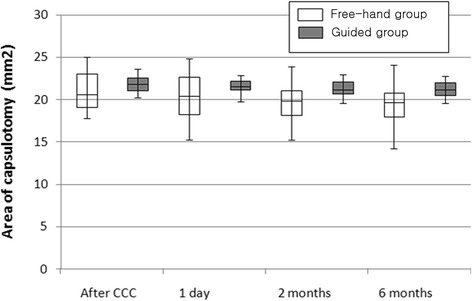
Area of capsulotomy at different time point: after CCC, postoperative day one, day two and six months. The box is determined by the central mean, the 35th percentile, and the 75th percentile. The whiskers are determined by the 5th and 95th percentiles

**Fig. 3:**
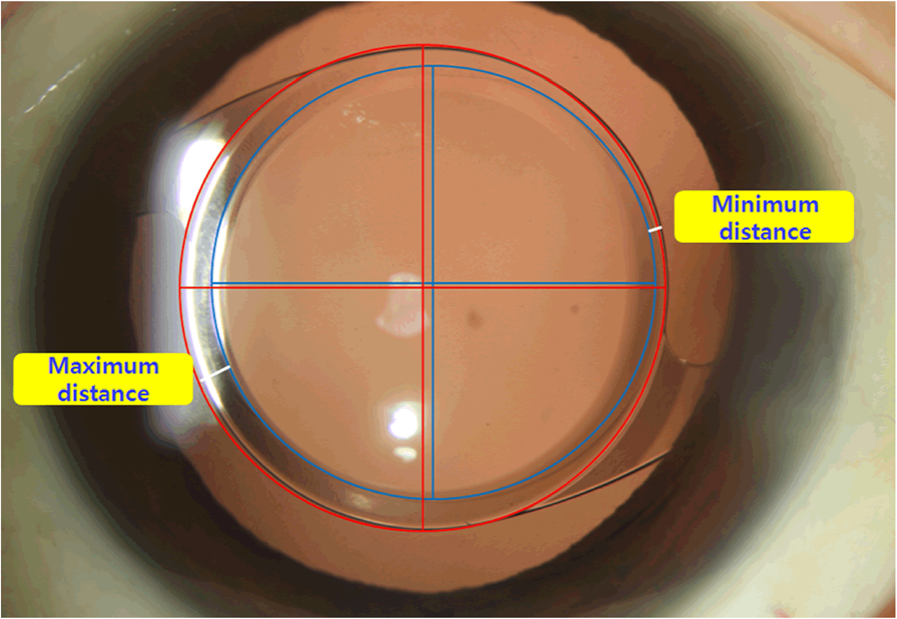
Maximum and minimum distances between the edge of the capsulotomy and the edge of the IOL optic were calculated to determine capsulotomy- and IOL- overlap

The change of capsulotomy area according to the time course are shown as box plots in Fig. 2. The guided group showed a larger capsulotomy area with little variation than that of the free-hand group. During the first two postoperative months, the circularity decreased significantly in the free-hand group; however, no significant changes in circularity were found in the guided group (*P* = 0.047). Figure 3 shows a case of ideal capsulorrhexis, taken on postoperative day 1, illustrating complete overlap of the edge of the IOL optic.

## Discussion

Manually constructing the anterior capsulorrhexis is technically challenging and is recognized as one of the most difficult aspects of cataract surgery to learn [15]. Free-hand capsulorrhexis is complicated by capsular tears in approximately 1% of cases [16]. Methods to improve manual capsulorrhexis using physical or virtual calipers have been developed [8, 10, 17]. However, all of these methods have their limitations. For example, Wallace’s capsulotomy diameter mark, a reference ring that projects through the light source of the microscope to the anterior capsule, provides only a rough guidance. They tend to be affected by magnification or distortion caused by the cornea. The ring-shaped caliper proposed by Tassignon et al. can be deformed or damaged during insertion and removal. VERUS ophthalmic caliper has wider width comparing the ORGC which makes difficult to be used in patients with small pupil size.

Recently introduced femtosecond laser technology enables surgeons to create more uniform, accurate, and predictable anterior capsulotomy than that produced by manual capsulorrhexis [5, 16, 18–20]. Despite its perceived benefits, femtosecond laser-assisted cataract surgery is not widely used, even in high-volume refractive centers. This is largely due to the significant financial costs involved in its implementation [21].

Our results suggest that the guider helps a capsulorrhexis to have a good circularity and closer to the target diameter 5.3 mm. Although the horizontal diameter was not significantly different between the two groups, the vertical diameter in the guided group was significantly closer to the target diameter than in the free-hand group. There was a greater degree of variability in the free-hand group, as evidenced by the different standard deviations, for both area and circularity compared to the guided group.

For free-hand capsulorrhexis, the size of the CCC could potentially have a smaller vertical area because this technique requires an uncomfortable pivot movement of the capsulotomy forceps through the small clear corneal incision site.

The accuracy and circularity of the capsulotomy size created by the guider were better at all time points measured after surgery. The CCC size decreased over time in both groups because of capsular contraction, although it was not statistically significant. Interestingly, the circularity of capsulorrhexis decreased significantly in the first 2 months postoperatively, only in the free-hand group. On the other hand, guided group maintained its circularity for postoperative 6 months.

We also analyzed the rate of ideal capsulorrhexis in two groups. With guider use, the surgeon can choose the exact location of the guider according to the limbus or dilated pupil center. In this study, the surgeon tried to place the guider between the center of limbus and dilated pupil, so the ideal capsulorrhexis rate was higher than free-hand group which was centered to the dilated pupil.

The major advantage of ideal capsulorrhexis is that it provides full control of lens epithelial cell proliferation, preventing posterior capsule opacification (PCO). Although we did not investigate the incidence of after-cataract in this study, this technique is expected to reduce after-cataract.

Through evaluation of the maximum and minimum distances between the IOL optic and CCC edge, we found that there were no differences across the two groups. This observation may be because of two reasons. First, the size of CCC is much smaller in the free-hand group. In the free-hand group, both maximum and minimum distances were larger than guided group’s distances. Second, if an ideal capsulorrhexis is made in the exact center of the capsular bag, the difference between maximum and minimum distances might be close to zero. These results mean that our capsulorrhexis was not made at real center of the lens capsule. Further study should be performed about the effect of using an ORGC guider on IOL centration.

A limitation of our study is that we analyzed patient charts retrospectively, which could have introduced selection bias. Although we used several IOL types, we selected only one IOL type to control the IOL’s influence on the results.

## Conclusions

We believe that the open ring-shaped guider for continuous curvilinear capsulorrhexis is a convenient and inexpensive tool that facilitates perfect capsulorrhexis shape and size and optimizes the outcome 6 months after the surgery.

## Abbreviations

CCC: Continuous curvilinear capsulorrhexis
ELP: Effective lens position
IOL: Intraocular lens
ORGC: Open ring-shaped guider for CCC
PCO: Posterior capsule opacification
